# Comparative Susceptibility of Madin–Darby Canine Kidney (MDCK) Derived Cell Lines for Isolation of Swine Origin Influenza A Viruses from Different Clinical Specimens

**DOI:** 10.3390/v13122346

**Published:** 2021-11-23

**Authors:** Matthew Suderman, Mariko Moniwa, Tamiru N. Alkie, Davor Ojkic, Andre Broes, Neil Pople, Yohannes Berhane

**Affiliations:** 1 National Centre for Foreign Animal Disease, Canadian Food Inspection Agency, Winnipeg, MB R3E 3R2, Canada; matthew.suderman@inspection.gc.ca (M.S.); mariko.moniwa@inspection.gc.ca (M.M.); tamiru.alkie@inspection.gc.ca (T.N.A.); 2 Animal Health Laboratory, University of Guelph, Guelph, ON N1G 2W1, Canada; dojkic@uoguelph.ca; 3 Biovet Inc., Saint-Hyacinthe, QC J2S 8W2, Canada; abroes.broes@biovet-inc.com; 4 Veterinary Diagnostic Services, Manitoba Agriculture and Resource Development, 545 University Crescent, Winnipeg, MB R3T 5S6, Canada; Neil.Pople@gov.mb.ca; 5 Department of Animal Science, University of Manitoba, Winnipeg, MB R3T 2N2, Canada; 6 Department of Veterinary Pathology, Western College of Veterinary Medicine, University of Saskatchewan, Saskatoon, SK S7N 5B4, Canada

**Keywords:** Madin–Darby canine kidney (MDCK) cells, virus isolation, swine influenza, nasal swabs, oral fluids

## Abstract

Madin–Darby canine kidney (MDCK) cells are commonly used for the isolation of mammalian influenza A viruses. The goal of this study was to compare the sensitivity and suitability of the original MDCK cell line in comparison with MDCK-derived cell lines, MDCK.2, MDCK SIAT-1 and MDCK-London for isolation of swine-origin influenza A viruses (IAV-S) from clinical specimens. One-hundred thirty clinical specimens collected from pigs in the form of nasal swabs, lung tissue and oral fluids that were positive by PCR for the presence of IAV-S RNA were inoculated in the cell cultures listed above. MDCK-SIAT1 cells yielded the highest proportion of positive IAV-S isolations from all specimen types. For nasal swabs, 58.62% of the specimens were IAV-S positive in MDCK-SIAT1 cells, followed by MDCK-London (36.21%), and conventional MDCK and MDCK.2 cells (27.5%). For lung specimens, 59.38% were IAV-S positive in MDCK-SIAT1 cells, followed by MDCK-London (40.63%), and conventional MDCK and MDCK.2 cells (18.75–31.25%). Oral fluids yielded the lowest number of positive virus isolation results, but MDCK-SIAT1 cells were still had the highest rate (35%) of IAV-S isolation, whereas the isolation rate in other cells ranged from 5–7.5%. Samples with lower IAV-S PCR cycle threshold (Ct) values were more suitable for culturing and isolation. The isolated IAV-S represented H1N1-β, H1N2-α, H1N1pdm and H3N2 cluster IV and cluster IVB viruses. The result of the current study demonstrated the importance of using the most appropriate MDCK cells when isolating IAV-S from clinical samples.

## 1. Introduction

Swine-origin influenza A viruses (IAV-S) cause a respiratory disease in pigs which is often complicated by infection with secondary bacterial respiratory pathogens [1]. The zoonotic potentials of IAV-S are also concerning [2]. Three subtypes of IAV-S, namely H1N1, H1N2 and H3N2 viruses have been isolated from pigs worldwide [3,4] and continue to evolve genetically and antigenically in swine herds [2,3]. The first evidence of isolation of influenza A viruses (IAVs) from pigs dates back to 1930 [5]. Since then, embryonating chicken eggs (ECE) and mammalian and avian origin primary and continuous cells have been used for the recovery of IAV-S from pigs [6,7,8,9,10,11,12]. While virus isolation in embryonated chicken eggs and many molecular techniques for IAV-S detection are available, isolation and characterization of IAV-S in continuous cell culture systems has some advantages and additional benefits [13].

Generally, IAVs have different cell receptors for entry into susceptible cells [14] and the host proteases required for the cleavage of the hemagglutinin (HA) protein to render the virus infectious differ [15,16]. Thus, IAV may express different replication properties in vitro and in vivo. A critical point for the primary isolation of IAV-S from complex clinical specimens is the availability of a cell line supporting isolation with minimal or lack of genetic mutations introduced by adaptation to the cell culture system [17,18]. It has been reported that A/H3N2 isolates cultured on conventional MDCK often acquire mutations that can result in amino acid changes in the HA and NA glycoproteins [18,19]. Additionally, genetic mutations in the HA protein and frequent re-assortment that introduced genetic and antigenic diversity affect the cellular tropism and replication of IAV in conventional MDCK cell lines [20,21,22,23].

MDCK cells are considered universal and sensitive cells for primary isolation of IAV-S from clinical specimens [24,25,26] since its establishment in 1958 from a kidney of a healthy cocker spaniel dog [27]. They support the replication of many avian, human and swine origin IAVs because they express two cell surface receptors with sialyloligosaccharides containing terminal N-acetyl sialic acid linked to galactose either by an α2,6-linkage (NeuAcα2,6Gal) or α2,3-linkage [28]. While human and predictably swine IAVs prefer to bind to NeuAcα2,6Gal present on the surface of MDCK cells, avian origin IAVs bind to NeuAcα2,3Gal to enter and replicate in permissive cells [14,29,30]. The upper respiratory epithelial cells in humans and swine as targets of influenza virus infection and replication express mostly of α-2,6-sialoglycans and less of α-2,3-sialoglycans [31,32,33]. MDCK cells modified to mimic the upper respiratory tract in terms of the density of the sialic acid receptors may improve the replication of mammalian origin IAVs instead of avian origin IAVs [18]. Therefore, to increase the susceptibility of MDCK cells to mammalian origin IAVs MDCK cells were modified to express higher amounts of NeuAcα2,6Gal and allow for more efficient replication of human IAVs [34,35]. Previous studies have demonstrated that MDCK-SIAT1 cells were more susceptible to the isolation of H1N1 directly from human clinical specimens compared to conventional MDCK cells [36]. All H1N1 viruses that grew in this modified cell line retained hemagglutinating properties. Although H3N2 viruses grew well in this cell line, only half of them retain the properties of hemagglutinating avian RBCs [36]. The progeny A/H3N2 viruses obtained from modified MDCK cells are still genetically stable [18]. The objective of the current study was to compare the susceptibility of conventional MDCK cell line and MDCK-derived cell lines for the primary isolation of IAV-S from various clinical specimens such as nasal swabs, lung samples and oral fluids that were collected from Canadian swine herds.

## 2. Materials and Methods

### 2.1. Clinical Samples

One-hundred-and-thirty clinical specimens in the form of nasal swabs (*n* = 58), lung tissues (*n* = 32) and oral fluids (*n* = 40), from swine that were positive for the presence of IAV-S genomic material based on the results of matrix gene based real-time RT-PCR were submitted to the National Centre for Foreign Animal Disease (NCFAD, Winnipeg, Manitoba) by the Animal Health Laboratory (University of Guelph, Guelph, Ontario), Veterinary Diagnostic Services (Winnipeg, Manitoba Agriculture and Resource Development) and Biovet Inc (Saint-Hyacinthe, QC, Canada).

### 2.2. Cells

MDCK (CCL-34; ATCC) and MDCK.2 (CRL-2936; ATCC) cell lines were obtained from the American Type Culture Collection (ATTC, Manassas, VA, USA). MDCK-SIAT1 (05071502; ECACC) was obtained from the European Collection of Authenticated Cell Cultures (ECACC, Salisbury, UK). MDCK-London cells were kindly provided by Dr Todd Davis (Communicable Diseases Center, Atlanta, GA, USA). MDCK, MDCK-SIAT1 and MDCK-London cells were passaged in Dulbecco’s Modified Eagle’s Medium (DMEM, Sigma, St Louis, MO, USA) supplemented with 10% fetal bovine serum (FBS) and 1× *GlutaMAX* (Thermo Fisher, Grand Island, NY, USA). The MDCK-SIAT1 cells were further supplemented with 2 mg/mL G418 (Gibco, Geneticin). MDCK.2 cells were passaged with minimum essential medium (MEM, Corning) supplemented with 10% FBS, 1× *GlutaMAX*, 1× non-essential amino acids and 1% sodium pyruvate. The virus infection medium for virus isolation includes 2% BSA for all cells in their respective basal media and G418 was removed from the MDCK-SIAT1 cells.

### 2.3. Virus Isolation

MDCK and MDCK.2 cells were seeded at a density of 4.2 × 10^5^ cells/well in a 12 well tissue culture plates (Costar, NY, USA). MDCK-SIAT1 and MDCK-London cells were seeded at 2.3 × 10^5^ cells/well in a 12 well tissue culture plates. The cells were incubated at 37 °C and 5% CO_2_. The next day plates were washed once with Ca^+^/Mg^+^ free PBS (Multicell, St. Bruno, QC, Canada). Five-fold diluted (500 µL/well) clinical specimens from nasal swabs, lung homogenates and oral fluids in DMEM containing a mixture of streptomycin (1 mg/mL), vancomycin (0.02 mg/mL), nystatin (50 U/mL) and penicillin 0.02 mg/mL) were added to the cell monolayer and allowed to adsorb for 1.5 h. The inoculum was removed from the cell monolayer and after three washes, 3 mL of virus infection media containing 0.67 µg/mL TPCK-trypsin (for MDCK, MDCK SIAT1 and MDCK-London cells) or 0.4 µg/mL TPCK-trypsin (for MDCK.2 cells) was added to the cell monolayer. The cells were then incubated at 37 °C and 5% CO_2_ and CPE were monitored daily for appearance of a cytopathic effect (CPE). The samples underwent a second passage in the same cells if no CPE was observed in the first inoculation [37]. After 3 days of incubation, cells with the media were freeze-thawed once and cell lysate were collected and centrifuged, and the presence of IAV-S was confirmed in the supernatant with real-time RT-PCR and hemagglutination activity (HA) was determined using a 0.5% turkey red blood cells as described previously [38].

### 2.4. RNA Extraction and Real-Time RT-PCR

The clinical specimens and cell lysate supernatants were clarified by centrifugation at 3000 rpm for 20 min at 4 °C. Total nucleic acids were extracted from 50 µL sample aliquots. Enterovirus RNA was added in the lysis buffer during RNA extraction process for monitoring the presence of PCR inhibitors in each sample. RNA extraction was carried out with the MagMAX™-96 total RNA isolation kit using the KingFisher 96-well robotic system (Applied Biosystems Ambion, Austin, TX, USA). The modified M1 gene specific real-time RT-PCR was used to confirm swine influenza A virus as described previously [39].

### 2.5. Hemagglutination and HI Assays and Genetic Analysis

Hemagglutinating activity of the cell lysate supernatants was determined following a two-fold serially diluted samples in 96-well V-bottom plates (Corning) as described elsewhere [38]. Turkey red blood cells (TRBC) at 0.5% concentration were added to assess hemagglutination activity. Hemagglutination inhibition (HI) assays were carried out for subtyping swine influenza A viruses present in the specimens and cell lysate supernatants. For this purpose, reference serum samples raised against H1N1/N2, H1N1pdm2009 and H3N2 were two-fold serially diluted in 96-well plates and to which 4 HA units containing cell lysate supernatant was added and incubated at room temperature. TRBCs at 0.5% was added and plates were further incubated for 30 min. Hemagglutination inhibition (HI) titers were determined as the reciprocal of the highest sample dilution resulting in complete inhibition of the RBCs from hemagglutination [38]. Moreover, sequencing and phylogenic analysis of HA gene was carried out to determine the genetic clades of the IAV-S isolates obtained from each cell lines as described in our paper [40].

### 2.6. Statistical Analysis

Descriptive statistics and proportions were used to summarize most of the data in this study. Student’s T-test statistics was used to analyze the HA data generated from isolates from nasal swabs in MDCK-SIAT1 and MDCK-London cells. A McNemar’s test statistic in a 2 by 2 table format was used to determine if there were differences of the effects of cell lines on the outcomes or detection of IAV-S isolates. Data were presented as mean ± standard error of the mean. A value of *p* < 0.05 was considered significant and chi-squared (χ^2^) value were provided when required.

## 3. Results

### 3.1. IAV-S Isolation from Clinical Specimens of Swine Origin

Virus isolation was attempted on all of 130 clinical specimens; 58 were nasal swabs, 32 lung samples and 40 oral fluid samples. All cell culture lysate supernatants were tested for IAV matrix gene using real-time RT-PCR to detect viral RNA and samples were confirmed positive for virus isolation when Ct values from lysates were lower than the original corresponding specimens. When some of the IAV-S isolates did not show typical hemagglutination properties in turkey RBCs and when CPE were atypical, samples from both passages were tested with qPCR for the presence of IAV-S genomic material. Clinical samples were considered positive for IAV-S when the Ct-values obtained from the cell culture were lower than the Ct-values of the original specimens. Generally, cytopathic effects (CPE) and HA titers were also considered for confirmation. The samples underwent a second passage in the same cells if no CPE was observed during first passage. When results from all the four cell lines were combined, IAV-S was isolated from 58.6% of nasal swabs as confirmed by real-time RT-PCR. For lung and oral fluids, IAV-S isolates were obtained from 65.63% and 35% of specimens, respectively (Table 1). Over all 19 lung samples were able to yield IAV-S isolates in one cell type and an additional 2 samples in another cell line, so a total of 21 lung samples were able to yield IAV-S isolates (Table 1).

### 3.2. Comparative Susceptibility of MDCK Cell Lines for IAV-S Isolation

For the comparative susceptibility study, 58 nasal swabs were propagated on MDCK-SIAT1 cells and 34 (58.6%) of the samples were able to yield IAV-S isolates. Similarly, in MDCK-London cells, 36.2% (21 out of 58) of these nasal swabs yielded IAV-S isolates. For the nasal swabs, using MDCK.2 and conventional MDCK cells, 27.5% (16 out of 58) of the samples were positive on isolation in each cell line. Among the lung samples tested, 59.38% (19 out of 32) generated IAV-S when propagated on MDCK-SIAT1 cell line and 40.6% (13 out of 32) of these samples were found positive on isolation when propagated on MDCK-London cells. When MDCK.2 cells were compared with conventional MDCK cells for isolation of IAV-S from lung specimens, the isolation rate for IAV-S was higher in MDCK.2 cells (31.25%) compared to conventional MDCK cells (18.7%). For oral fluid specimens, MDCK-SIAT1 was found most suitable for virus propagation compared to other cell lines used. In this case, 35% of oral fluid samples yielded positive isolation results on MDCK-SIAT1 cells, whereas for MDCK-London, MDCK.2 and conventional MDCK, the IAV-S isolation rates ranged from 5–7% (Table 2).

### 3.3. Comparative Statistical Analyses Using the McNemar’s Test

The McNemar’s test analysis was focused on data obtained from nasal swabs and lungs only as oral fluid specimens yielded a small number of IAV-S isolates in the other three cell lines (MDCK-London, MDCK.2 and MDCK cells) used in this study. The McNemar’s test was performed in a 2 by 2 table format and was used to determine if there were differences in the sensitivity of the cell lines on IAV-S isolation (Table 3).

With regard to nasal swabs, of the 34 samples producing IAV-S isolates on MDCK-SIAT1 cells (Table 2), 13 of them did not yield IAV-S isolates on MDCK-London; similarly, 18 of them were isolation negative on both MDCK.2 and MDCK cells (Table 3) indicating MDCK-SIAT1 cells were more sensitive in isolating IAV-S compared to MDCK-London (χ^2^ = 13, *p* = 0.0002), and MDCK.2 and MDCK (χ^2^ = 18, *p* < 0.05) cells. In MDCK-SIAT1 cells, 19 lung samples (Table 2) were positive on isolation, however, 8 of these samples did not yield isolates when propagated on MDCK-London. Similarly, 9 and 13 of them were found isolation negative when propagated on MDCK.2 and MDCK cells, respectively (Table 3). These results indicate that MDCK-SIAT1 cells were more sensitive for IAV-S isolation over MDCK-London (χ^2^ = 3.6, *p* = 0.0578), MDCK.2 (χ^2^ = 9, *p* = 0.0039) and MDCK (χ^2^ = 13, *p* = 0.0002) from the lung specimens. The MDCK-SIAT1 cell line was the most sensitive substrate for isolating IAV-S. All clinical samples that did not yield isolates on MDCK-SIAT1 cell line were also negative on other cells, except that two lung samples that were isolation positive on MDCK-London cells.

Regarding nasal swabs, of 21 samples that were confirmed positive on MDCK-London (Table 2), 7 and 6 samples were isolation negative when propagated on MDCK.2 and MDCK cells respectively (Table 4) indicating that MDCK-London was marginally better in isolating IAV-S compared to MDCK.2 (χ^2^ = 2.78, *p* = 0.09), and MDCK (χ^2^ = 3.57, *p* = 0.06) cells. In MDCK-London, of the 13 lung specimens showing positive isolation results for IAV-S (Table 2), 5 and 7 of them were isolation negative when propagated on MDCK.2 and MDCK cells, respectively (Table 4). The results indicated that MDCK-London and MDCK.2 had no significant differences in the isolation of IAV-S (χ^2^ = 1.29, *p* = 0.45), whereas MDCK-London was relatively better in yielding more isolates than MDCK cells (χ^2^ = 7, *p* = 0.0156) from lung specimens. MDCK.2 and conventional MDCK cells yield fewer positive isolates and were the least preferred cell lines for isolating IAV-S from lung samples, nasal swabs and oral fluids (Table 2 and Table 4).

### 3.4. Effects of Clinical Specimen PCR Cycle Threshold (CT) Values on the Outcomes of IAV-S Isolation

To evaluate the chance of isolating IAV-S from nasal swabs, lung tissues, and oral fluids on four different MDCK cell lines, we screened and categorized the clinical samples as having Ct values of lower than 20, 21–25 or higher than 26. This classification was based on the results of real-time RT-PCR targeting the matrix gene of IAV-S in the original clinical specimens.

With lung specimens, as most of the samples had Ct values of lower than 25, 18 out of 29 (62.1%) of these samples yielded virus isolates in MDCK-SIAT1 cell line, 13 out of 29 (44.8%) in MDCK-London, 10 out of 29 (34.5%) in MDCK.2 and 6 out of 29 (20.7%) in MDCK cell lines (Table 5). The trend of isolating IAV-S from nasal swabs and oral fluids was similar to the results described for lung specimens whereby most samples with Ct values of <25 yielded IAV-S isolates. Lung, nasal swabs, and oral fluids with >26 Ct values were either isolation negative or only few samples yielded positive isolates. Normally, clinical specimens with lower PCR Ct-values were more likely to yield IAV-S isolates when propagated on MDCK cell derivatives.

### 3.5. Determining HA Activity and Subtyping of IAV-S Isolates

**Figure 1:**
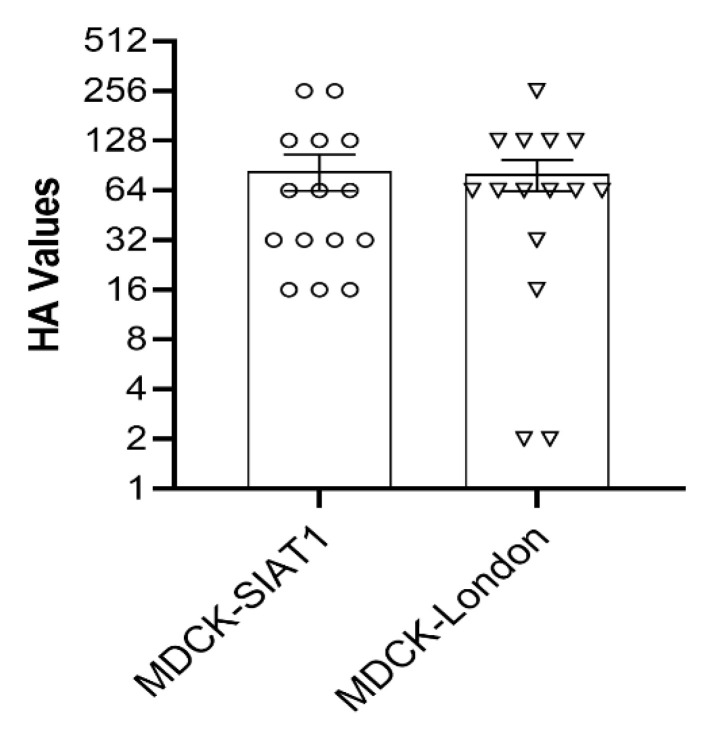
HA titers of IAV-S isolates from nasal swabs propagated on MDCK-SIAT1 and MDCK-London cells.

Hemagglutination assay was used to determine the presence or absence of hemagglutinating virus in all cell lysate supernatants for head-to-head comparison of HA titers, we used IAV-S isolates acquired from nasal samples in MDCK-SIAT1 and MDCK London cells only. The HA titers of IAV-S isolates obtained from nasal swabs that were propagated in the same way on MDCK-SIAT-1 and MDCK-London cell lines showed significantly higher HA titers compared to the remaining cell lines. Our results demonstrated that the HA titers were comparatively similar in both cell lines (Figure 1, *p* < 0.6), although some IAV-S isolates collected from MDCK-SIAT1 cells had higher HA titers.

Except for two lung samples that were positive for IAV-S on MDCK-London cells all other clinical samples that did not yield IAV-S isolates on MDCK-SIAT1 cell line were also negative on other MDCK cell lines. All virus isolates that turned positive for IAV matrix gene by real-time RT-PCR were subtyped and their HAs sequenced to ascertain the frequency of occurrence of the different IAV subtypes and their designated clades. The isolated IAV-S subtypes from all clinical samples included H3N2 IV, H3N2 IVB, H1N1pdm, H1N1β and H1N2α. As indicated on Table 6, the proportions of H3N2 IV (23.5%), H3N2 IVB (17.6%), H1N1pdm (17.6%) H1N1β (8.8%) and H1N2-α (32.4%) that were isolated using MDCK-SIAT1 from nasal swabs were almost comparable, this probably indicate that MDCK-SIAT1 can be suitable and equally susceptible to infections with different subtypes/clades of IAV-S. However, controlled experimental infections using standard virus doses in MDCK-SIAT1 cells are required for reaching a conclusion that this cell line equally supports the replication of selected subtypes of IAV-S so as to introduce it for routinely propagating and isolating viruses originated from clinical cases of IAV-S. In the lung and oral fluids, although the overall proportions of each subtype and clade are quite smaller, the data suggested that IAV-S subtypes may not affect the success of isolation on MDCK-SIAT1 cells (Table 6).

## 4. Discussion

Swine-origin influenza A viruses (IAV-S) replicate in a range of primary and continuous cells of mammalian and avian origins [6,7,8,9,10,11]. Nevertheless, many IAV diagnostic and research labs prefer MDCK cells, which are composed of a heterogeneous cell population [36] that express different levels of sialic acid receptors that in turn influence susceptibility to IAV infection [41]. In the current study, we presented the results of the susceptibility of four different MDCK cells for isolation of IAV-S from different clinical specimens.

Nasal swabs and lung specimens yielded more IAV-S isolates compared to oral fluid samples. Still, close to half of the nasal swabs, lung and oral fluid specimens did not yield positive isolation results on cell culture, although they tested positive for the presence of IAV-S RNA with real-time RT-PCR that targeted the matrix gene of the virus. Although oral fluids from pigs experimentally infected with influenza viruses were positive on real-time RT-PCR, virus isolation in MDCK cells was not successful [42]. Similarly, in our study most of the oral fluids with lower PCR Ct values did not yield virus isolates compared to nasal and lung samples. This could be a result of inactivation of the virus by enzymes and other substances that may be present in the saliva or the environment that have negatively impacted viability of the viruses.

MDCK-SIAT1 was the most susceptible cell line that supported the replication of larger number of IAV-S isolates from nasal swabs, lung tissue as well as oral fluids. MDCK-London, followed by MDCK.2 cell line also performed better for IAV-S isolation than conventional MDCK cells, which was the least susceptible. A recent study published similar observations whereby selected subtypes of human IAVs with standard infectious titers replicated efficiently on MDCK-SIAT1 cells [36]. Previous works compared the sensitivity of MDCK-London and MDCK-SIAT1 cell lines and demonstrated that MDCK-London cells are better suited for the propagation of human IAVs such as epidemic H1N1, pandemic H1N1 and seasonal H3N2 viruses that were preadapted to growing in embryonated chicken eggs [43]. Furthermore, viruses grown in MDCK-London cells had comparatively higher infectious virus titers, HA titers and NA activity [43,44]. Although HA titers may not accurately reflect the quantity of infectious viruses, it rapidly enables virus quantification. In our study, irrespective of the subtypes of IAV-S, the HA titers remained similar on both MDCK-London and MDCK-SIAT1 cells indicating viruses grown on both cells maintain functional competence of HA protein to hemagglutinate avian RBCs. AX4, another engineered cell line derived from MDCK cells performed better than conventional MDCK cell line for propagation of IAVs originated from clinical human samples and also for lab grown seasonal human IAV using a standard infectious dose [34,35,36]. Another “humanized” MDCK cell line called hCK has been tested effective for the primary isolation of human A/H1N1pdm and A/H3N2 viruses [18]. In a comparative study using MDCK cells, AX4 and hCK, it has been shown that A/H1N1pdm viruses replicated to significantly higher titers in all cells. However, A/H3N2 viruses replicated to higher titers only in hCK cells [18]. Other recent studies concluded the relatively lower sensitivity of conventional MDCK cells to most recent A/H3N2 viruses than engineered cells [34,36,45].

The extent of human origin IAV replication is known to correlate with the density and type of the sialic acid-receptors on susceptible host cells. In this regard, MDCK-SIAT1 cells used in our study and AX4 cells express more human type α-2,6-galactose linked sialic acid than α-2,3-galactose linked sialic acid through genetic modifications [34,35]. On the other hand, the humanized MDCK cells (hCK) overexpressed higher levels of α-2,6-sialoglycans, with suppressed expression of the natural α-2,3-sialoglycans. MDCK-London (FR-58) is a non-engineered cloned cell derived from MDCK cell line and express moderate levels of both receptors at comparable levels [43]. Conversely, conventional MDCK cells constitutively express relatively low levels of both sialic acid receptors [43]. Generally, while conventional MDCK cells are less efficient for IAV replication, the engineered and cloned cells showed greater susceptibility to mammalian origin influenza viruses, particularly to human subtypes [25,26]. The isolation of large number of primary IAV-S isolates in MDCK-SIAT1 without serial passage is critical for genetic and antigenic profiling of IAV-S. Mostly, specimens that are serially passaged to increase the chance of virus recovery, likely introduces adaptative mutations. For example, human origin subtype A/H3N2 viruses cultured using conventional MDCK cell line has been shown to undergo adaptative mutations on surface glycoproteins [19]. Therefore, we present in this study that MDCK-SIAT1 and MDCK-London are valuable cell culture systems for isolating IAV-S.

Virus isolation in cell culture requires, screening of clinical samples for IAV-S using real-time RT-PCR. As the cycle threshold (Ct) values correlate with virus load in the samples or with the quantity of genetic materials, those samples with lower Ct values are preferred for virus isolation in cell culture [46]. Most specimens in the current study had lower Ct values (<25) for IAV-S matrix gene indicating more viral RNA or viral load in the clinical specimens. As expected, most of the nasal swabs with lower Ct values were positive for IAV-S isolation from MDCK cell lines. Nasal swabs with Ct values > 26 (correspond to lower genetic materials or viral load) yielded mostly negative IAV-S isolation on MDCK.2 and MDCK cells, showing that conventional MDCK cell lines are not sensitive to lower infectious titers. However, still a large number of nasal swabs with higher Ct values gave positive IAV-S isolation results in MDCK-SIAT1. On the other hand, as indicated previously [42,47] oral fluids were least suitable for IAV-S isolation rate despite their low Ct values. Using antigenically related human IAVs (H3N2 and H1N1 subtypes), it was shown that low virus titers did not yield virus growth on conventional MDCK cells, however viral growth was successful on engineered MDCK cells [34]. When higher infectious doses of human H3N2 and H1N1 viruses were used, conspicuous virus growth was observed on both conventional MDCK cells and engineered cells. However, the resulting virus titers from MDCK-SIAT1 was by far higher than from conventional MDCK cells [34]. Some isolates of human H3N2 viruses propagated in embryonated chicken eggs (ECE) could reach to the same titer on both conventional MDCK cell line and modified cells which may be due to adaptation changes of ECE-propagated viruses [34].

The impacts of genetic diversity [14] and the subtypes of IAV on the outcomes of virus isolation on cell substrate have been documented. In the current study, even if we did not address this aspect directly using a controlled experiment, the proportions of A/H3N2, A/H1N1pdm and A/H1N2-α isolated using MDCK-SIAT1 from nasal swabs were almost comparable indicating the suitability of MDCK-SIAT1 for IAV-S isolations of various subtypes. Similar trends were observed for lung and oral fluid specimens in isolating IAV-S. In our study, close to 40% of the nasal swabs, 35% of lung samples and 65% of oral fluid were negative for IAV-S when propagated on MDCK-SIAT1 even if the original samples were regarded as positive for IAV-S based on screening by RT-PCR. The success rates of isolation of IAV-S in MDCK-SIAT1 were lower when compared to the higher efficiency studies in humans, where all human specimens with confirmed A/H1N1pdm and A/H3N2 viruses by real-time RT-PCR were isolated on a humanized MDCK cell, called hCK [18]. From human clinical specimens, 90% of A/H3N2 and all A/H1N1 viruses replicated on AX4, another engineered MDCK cell resembling MDCK-SIAT1. Over 80% of H3N2 and all A/H1N1 grew on conventional MDCK cells [18]. However, the nature of the clinical specimens whereby both H3N2 and A/H1N1 were obtained, and the actual Ct values of the specimens were not stated in this study. In addition, the study on conventional MDCK cells used mostly the HA gene to confirm the presence of IAV in the human clinical samples as opposed to the matrix gene used in our study [18]. Generally, MDCK-SIAT1 cells support the replication of most human origin IAVs as stated previously and also in our study significant proportions of IAV-S replicated on MDCK-SIAT1 cells.

## 5. Conclusions

The study revealed that there was a difference between MDCK-derived cell lines in supporting the replication of IAV-S. Although many laboratories use conventional MDCK cells for routine IAV isolation for diagnostic and research purposes our study demonstrated the poor efficacy of this cell line. MDCK-SIAT1 and MDCK-London were more sensitive and suitable for isolation IAV-S from various clinical specimens and were better alternatives to conventional MDCK cells. The data from our study also highlighted the Ct values of the original specimens as the major determinant of positive isolation results. Oral fluids appeared to be the least suitable sample type, but even for oral fluids the proportion of isolation in MDCK-SIAT1 cells was higher than in other cell lines. The isolation of large number of IAV-S isolates in MDCK-SIAT1 without serial passages is critical for genetic and antigenic profiling of IAV-S.

## Figures and Tables

**Table 1:** Total number of IAV-S isolates obtained when a combination of MDCK cell lines were used to propagate the virus from swine origin clinical specimens.

Specimen Types	Total Tested (*n*)	Total Confirmed Positive by PCR (*n*)	Proportion Positive (%)
Nasal swab	58	34	58.6
Lung	32	21	65.63
Oral fluid	40	14	35

**Table 2:** Comparative susceptibility of MDCK cell lines for IAV-S isolation based on the types of clinical specimens.

Specimens	Tested	MDCK-SIAT1		MDCK-London		MDCK.2		MDCK	
		+ve	% +ve	+ve	% +ve	+ve	% +ve	+ve	% +ve
Nasal swab	58	34	58.62	21	36.21	16	27.58	16	27.58
Lung	32	19	59.38	13	40.63	10	31.25	6	18.75
Oral fluid	40	14	35	2	5	3	7.5	3	7.5

**Table 3:** Performance of MDCK-SIAT1 cell compared with other three cell lines on the outcomes of IAV-S isolation.

Specimens	Tested in SIAT1	London+	London−	MDCK.+	MDCK.−	MDCK+	MDCK−
Nasal swab (*n* = 58)	SIAT1+ (34)	21	13	16	18	16	18
	SIAT1− (24)	0	24	0	24	0	24
Lung(*n* = 32)	SIAT1+ (19)	11	8	10	9	6	13
	SIAT1− (13)	2	11	0	13	0	13

**Table 4:** Performance of MDCK-London compared with MDCK.2 and MDCK cells lines, and MDCK compared with MDCK.2 on the outcomes of IAV-S isolation.

Samples	Tested in London (*n*)	MDCK.2 +	MDCK.2 −	MDCK+	MDCK −	Tested in MDCK (*n*)	MDCK.2+	MDCK.2 −
Nasal swab (*n* = 58)	London+ (21)	14	7	15	6	MDCK+ (16)	14	2
	London− (37)	2	35	1	36	MDCK − (42)	2	40
Lung (*n* = 32)	London+ (13)	8	5	6	7	MDCK+ (6)	5	1
	London− (19)	2	17	0	19	MDCK − (26)	5	21

McNemar’s test compared each time the performance of two cell lines at a time for each specimen. The outcomes were reported as either positive for virus isolation or negative for virus isolation and the test provided a Chi-squared (χ^2^) value.

**Table 5:** The effects of original specimen PCR cycle threshold (Ct) values determine the outcome of IAV-S isolation in four cell lines.

Samples	Ct Values of Original Samples	Total Tested (*n*)	SIAT1 (*n*, %)	London (*n*, %)	MDCK.2 (*n*, %)	MDCK (*n*, %)
Nasal swab	<20	8	6 (75)	6 (75)	5 (62.5)	3 (37.5)
	21–25	33	24 (72.7)	15 (45.5)	11 (33.3)	13 (39.4)
	>26	17	4 (23.5)	0 (0)	0 (0)	0 (0)
Lung	<20	19	12 (63.2)	10 (52.6)	7 (36.8)	5 (26.3)
	21–25	10	6 (60)	3 (30)	3 (30)	1 (10)
	>26	3	0 (0)	0 (0)	0 (0)	0 (0)
Oral fluid	<20	11	7 (63.6)	1 (9)	2 (18.2)	2 (18.2)
	21–25	15	4 (26.7)	1 (6.7)	1 (6.7)	1 (6.7)
	>26	14	3 (21.4)	0 (0)	0 (0)	0 (0)

**Table 6:** Proportions of IAV-S subtypes identified using a combination of cell lines.

Samples	+ve PCR	H3N2 IV		H3N2 IVB		H1N1pdm		H1N1-β		H1N2-α	
		+ve	%+ve	+ve	%+ve	+ve	%+ve	+ve	%+ve	+ve	%+ve
Nasal swab	34	8	23.5	6	17.6	6	17.6	3	8.8	11	32.4
Lung	18	1	5.6	4	22.2	4	22.2	1	5.6	2	11.1
Oral fluid	14	1	7.1	4	28.6	4	28.6	3	21.4	1	7.1

## Data Availability

All data generated and analyzed during this study are included in this article.
